# Profile of Heart Donors from the Human Valve Bank of the Santa Casa de Misericórdia de Curitiba

**DOI:** 10.5935/1678-9741.20160033

**Published:** 2016

**Authors:** Renata Maria Ferreira, Marise Teresinha Brenner Affonso da Costa, Osiris Canciglieri Junior, Ângelo Márcio Oliveira Sant'Anna

**Affiliations:** 1 Human Heart Valve Bank of the Santa Casa Misericórdia, Curitiba, PR, Brazil; 2 Pontifícia Universidade Católica do Paraná (PUCPR), Curitiba, PR, Brazil; 3 Universidade Federal da Bahia (UFBA), Salvador, BA, Brazil

**Keywords:** Heart Valves, Tissue Banks, Directed Tissue Donation, Statistical Analysis

## Abstract

**Introduction:**

Human heart valves are used as replacement valves and have satisfactory functional results compared with conventional prostheses.

**Objective:**

Characterize the profile of effective heart donors from the human valve bank of the santa casa de misericórdia de curitiba and analyze the association between the profile variables.

**Methods:**

It consists of a retrospective and quantitative study of electronic medical records from heart donors for heart valves. every heart donation made to the bank between january 2004 and december 2014 was studied.

**Results:**

2,149 donations were analyzed, from donors aged 0 to 71 years old, with an average of 34.9 ± 15.03 years old. most donors were male 65.7% (n=1,411) and 34.3% (n=738) were female. among the most frequent causes of the donors' death are trauma at 53% (n=1,139) and cerebral vascular accident at 34.2% (n=735). there was significant statistical association between the analyzed variables.

**Conclusion:**

There has been an improvement in brazil's donation rate, being essential that the tissue banks work together with the state and federal district centers for notification, procurement and distribution of organs in order to increase the number of donors.

**Table:** 

Abbreviations, acronyms & symbols	
BD	= Brain death
BVCH	= Human Valve Bank
CNCDO	= Centers for Notification, Procurement and Distribution of Organs
CPA	= Cardiopulmonary arrest
CRA	= Cardiorespiratory arrest
CVA	= Cerebrovascular accident
SNT	= National Transplant System

## INTRODUCTION

The progress of science and biotechnology has already revealed significant results, such as the possibility of new alternatives for human tissue transplantation, providing quality of life for victims of trauma and other degenerative diseases. Human tissue used for therapeutic purposes, such as cardiovascular tissue, skin, bone, cartilage, tendons, and corneas, are available from institutions known as Tissue Banks. Those institutions have physical facilities, equipment, human resources and techniques that enable the capture, processing, preservation, storage and distribution of human tissue from living donors or cadavers for allogeneic transplants^[1]^. Tissue Banks are responsible for ensuring tissue quality, receptor safety and maintenance of a satisfactory level for the transplant surgeons^[2]^.

Among cardiovascular tissue, human heart valves have been used as a replacement valve since 1962. When compared with conventional prostheses, valve grafts show satisfactory functional results, namely: physiological hemodynamic performance with central and laminar blood flow; almost zero incidence of thromboembolism, avoiding the use of anticoagulants; and high resistance to infections. Those specifications result in better quality of life for patients postoperatively, and, in some series, higher long-term survival^[3,4]^.

In Brazil, cardiovascular grafts are processed and made available by the Human Valve Bank of the Hospital de Caridade da Irmandade da Santa Casa de Misericórdia de Curitiba (BVCH). BVCH is a pioneer in the country and the only one in activity so far. It was founded in 1996 with the purpose of providing cardiovascular tissue for heart surgeries throughout the Brazilian territory. The bank initiative started due to the high prevalence of valvular replacements in a low socioeconomic population without access to adequate anticoagulant therapy^[4]^. In view of the importance of the BVCH work, this sudy aimed to characterize the effective donor profiles from the BVCH in the period between January 2004 and December 2014.

## METHODS

This is a retrospective study of electronic records of effective heart donors for valves, from January 2004 to December 2014, whose organs were received by BVCH. The chosen interval is justified due to a previous study^[4]^ which examined the activities of the BVCH eight initial years.

Heart retrieval for valves is held in most Brazilian states by teams registered in the Centers for Notification, Procurement and Distribution of Organs (CNCDO) and authorized by the National Transplant System (SNT). The donation process begins with the identification of the potential donor and family interview to offer them all necessary information and support regarding the decision to donate. All stages of the donation process are carried out respecting the ethical and legal principles of Law No. 9,434, of February 4, 1997^[5]^, which provides for the retrieval of organ and body tissue for transplantation and treatment. The law supports the CNCDO for organ retrieval without previous authorization from the hospital's ethics committee. It must be noted that, currently, organ retrieval in Brazil is carried out exclusively in hospital environments.

The family interview is conducted by the organ and tissue transplant services present at the hospital, in accordance with the CNCDO guidelines for organ retrieval or removal, and the BVCH does not take part in this stage of the process. In the past decade, obtaining permission from the family for organ and tissue retrieval was more difficult^[6]^, but recently this scenario has changed and the number of family permissions for donations has increased^[4]^. Nevertheless, the number of donations in Brazil is much lower compared to countries in Asia and Europe^[7,8]^.

Potential donors for heart valves are those in brain death (in circumstances in which the heart cannot be used for transplantation), in cardiopulmonary arrest (CPA), and heart transplant recipients. Hearts from transplant receivers with absence of valvulopathy are also accepted, depending on the clinical and quality inspection of the organ during the processing of tissue.

The organs must be transported following the specific protocol established by the BVCH, based on the Federal Decree 2600 of October 21, 2009^[1]^, preferably by air. The packaging used in the transport must be triple, sterile and waterproof, closed one by one. In the primary package, a transport solution (0.9% sodium chloride) is added in sufficient quantity to maintain the organ totally immersed, at temperature between 2°C and 8°C. The plastic packages containing the organ are accommodated in a rigid and airtight container, which is then placed in an isothermal box containing ordinary ice.

The information generated during the whole process, from the selection and screening of the donor to the final disposal of the cardiovascular tissue, is stored in physical and electronic means. The information available on the charts consists of sex, race, age, cause of the donor's death, city and state where the organ was retrieved.

Statistical analysis was performed using software SPSS 20 in order to characterize the profiles of heart donors for valves in the period from January 2004 to December 2014. The variables were described as frequencies and analyzed using Chi-square test, with statistical significance level of 5% (*P*<0.05).

## RESULTS

**Fig. 1:**
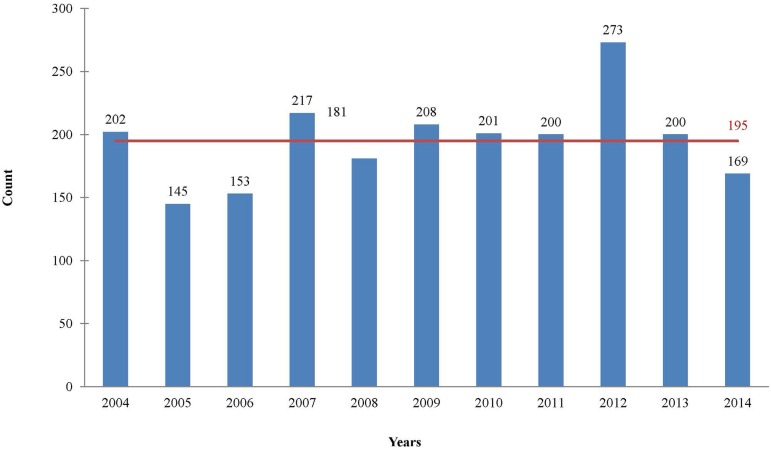
Distribution of retrieved hearts between 2004 and 2014.

**Fig. 2:**
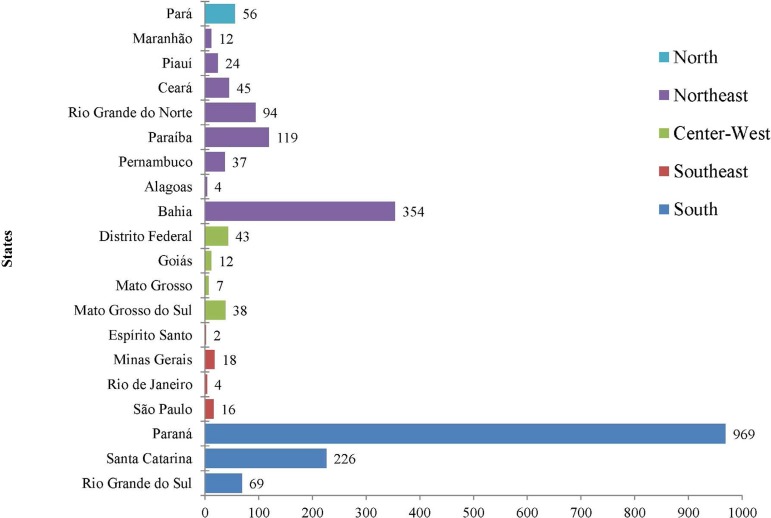
Distribution of retrieved hearts by State.

The BVCH received 2,149 donated hearts with an average ± standard deviaton of 195.36±34.65 hearts/year. It was observed that there has been a growth in the number of donations, except for a decline last year, as shown in Figure 1. The organs received were retrieved in 20 Brazilian States. However, the analysis of the frequency of retrieval by State showed a great discrepancy between them, as it can be seen in Figure 2.

**Fig. 3:**
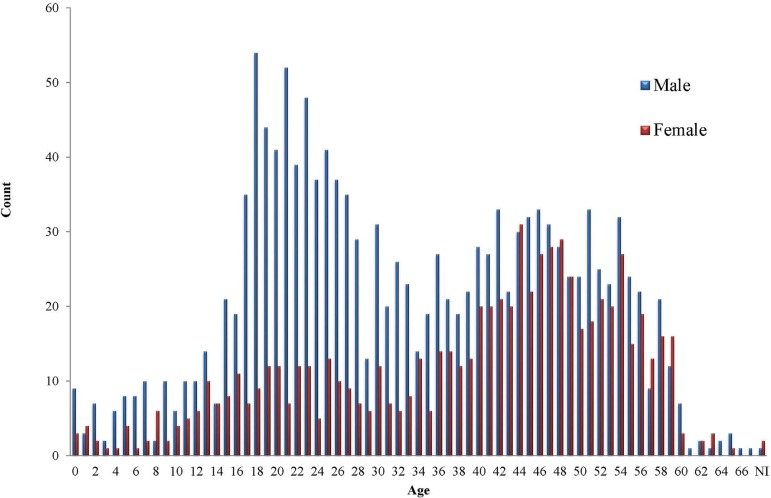
Distribution of heart donors by age and gender.

Concerning age, the youngest donor was less than 1 year old and the oldest was 71 years old, with an average age of 34.9±15.03 years. The age group of 0 to 15 years old represented a lower proportion of donors at 9.3% (n=199), the group of 16 to 35 years old represented 39.2% (n=841), a total of 33.2% (n=713) comprised the group of 36 to 50 years old, donors aged 51 or more represented 18.3% (n=393), and those whose age was not reported corresponded to 0.1% (n=3) of the total number of donors (Table 1). There was predominance of male donors in all age groups, 65.7% (n=1,410) *versus* 34.3% (n=738) of female donors. Figure 3 illustrates the distribution of age by gender.

**Table 1:** Distribution of heart donors by age.

Age	Gender				Total	
	Male		Female			
	n	%	n	%	n	%
0-15 years	133	6.2%	66	3.1%	199	9.3%
16-35 years	657	30.6%	184	8.6%	841	39.2%
36-50 years	401	18.7%	312	14.5%	713	33.2%
>51 years	219	10.2%	174	8.1%	393	18.3%
Total	1410	65.7%	736	34.3%	2146	100.0%

**Fig. 4:**
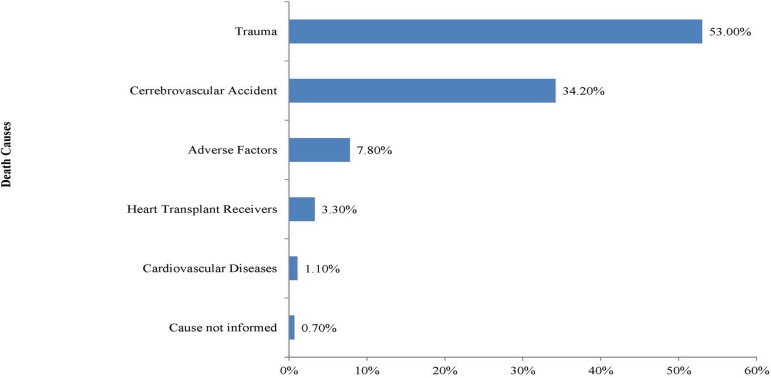
Distribution of donors according to causes of death.

The data analyzed showed that among the donors' cause of death, trauma was the most frequent at 53% (n=1,139), followed by cerebrovascular accident (CVA) with 34.2% (n=735), adverse factors to cardiology at 7.3% (n=167), cardiovascular diseases with 1.1% (n=24), and unreported causes at 0.7% (n=14). In addition, live donors (heart transplant receivers) accounted for 3.3% (n=70) of the total donations (Figure 4).

The age and cause of death variables were associated with gender and it was noticed the prevalence of male donors of 46.6% (n=657) in the group aged 16 to 35 years old and 42.4% (n=312) female donors in the group of 36-50 years old (*P*<0.001). Besides, it was observed that trauma represented more than half of the causes of death among male donors, with 68% (n=959), and CVA was responsible for with 59.8% (n=441) of deaths among female donors (*P*<0.001), as shown in Table 2.

**Table 2:** Association analysis for age, causes of death and gender.

	Gender		
	Male (n=1.410)	Female (n=736)	*P*-value
Age			
0-15 years	133 (9.4%)	66 (9.0%)	
16-35 years	657 (46.6%)*	184 (25.0%)	
36-50 years	401 (28.4%)	312 (42.4%)*	0.000
> 50 years	219 (15.5%)	174 (23.4%)*	
Causes of death			
Trauma	959 (68.0%)*	180 (24.4%)	
Cerebrovascular accident	294 (20.8%)	441 (59.8%)*	
Adverse factors	84 (6.0%)	83 (11.2%)*	0.000
Cardiovascular diseases	13 (0.9%)	11 (1.5%)	
Transplant receivers	50 (3.6%)	17 (2.6%)	
Not informed	10 (0.7%)	4 (0.5%)	

* Significant association based on the adjusted residual.

Causes of death were analyzed by age group and it was observed that trauma is significantly associated with donors between 16-35 years old, 81.2% (n=683), CVA was associated with donors in the 36-50 and >50 years old age groups, cardiovascular diseases and transplant receivers were associated with donors >50 years old (*P*<0.001), as stated in Table 3.

**Table 3:** Association analysis for age and causes of death.

	Age				
	0 - 15 (n=199)	16 - 35 (n=841)	36 - 50 (n=713)	> 50 (n=393)	*P*-value
Causes of death					
Trauma	114 (57.3%)	683 (81.2%)*	274 (38.4%)	68 (17.3%)	
Cerebrovascular accident	32 (16.1%)	98 (11.7%)	348 (48.8%)*	257 (65.4%)*	
Adverse factors	49 (24.6%)*	45 (5.4%)	47 (6.6%)	26 (6.6%)	0.000
Cardiovascular diseases	__	4 (0.5%)	9 (1.3%)	11 (2.8%)*	
Transplant receivers	3 (1.5%)	9 (1.1%)	31 (4.3%)*	26 (6.6%)*	
Not informed	1 (0.5%)	2 (0.2%)	4 (0.6%)	5 (1.3%)	

* Significant association based on the adjusted residual.

Regarding race, there was a preponderance of donors declared as white, 62.56% (n=1,334) of the total, while 22.60% (n=486) declared themselves as *pardo* and 9.12% (n=196) as black. An additional 5.72% (n=122) did not provide information relating to race in their records. Causes of death were also analyzed according to race and it was observed that trauma is significantly associated with donors declared as *pardo*, 57.6% (n=280), CVA was associated with black donors and transplant receivers with donors declared white (*P*<0.001), as shown in Table 4.

**Table 4:** Association analysis for race and causes of death.

	Race			
	White (n=1.345)	Black (n=196)	*Pardo* (n=486)	*P*-value
Causes of death				
Trauma	713 (53.0%)	90 (45.9%)	280 (57.6%)*	
Cerebrovascular accident	466 (34.6%)	88 (44.9%)*	152 (31.3%)	
Adverse factors	102 (7.6%)	12 (6.1%)	36 (7.4%)	0.000
Cardiovascular diseases	16 (1.2%)	1 (0.5%)	5 (1.0%)	
Transplant receivers	42 (3.1%)*	3 (1.5%)	11 (2.3%)	
Not informed	6 (0.4%)	2 (1.0%)	2 (0.4%)	

* Significant association based on the adjusted residual.

## DISCUSSION

Brazil stands out in the area of transplantation by owning one of the largest public programmes of organ and tissue transplantation in the world, working through a systematic and official protocol; however, a growing imbalance between supply and demand remains.

The deficiency of organ donation is a universal obstacle and the donor is the most important factor in the provision of tissue for transplantation^[9]^. The shortage of organ donors is not primarily the result of a lack of donors, but rather the difficulty of identifying them, getting the family's consent and retrieving the organ and/or tissue in a timely manner. In this context, intra-hospital transplant committees are essential in identifying the potential donor and promptly notifying the State Transplant Centers^[6]^.

The evolution of patients with brain death (BD) to cardiorespiratory arrest (CRA) was one of the main causes of non-completion of the donation of solid organs for transplantation in 2012, reaching 52% in some Brazilian regions^[10]^. It is worth mentioning that for donation of a solid organ for transplantation the potential donor should necessarily have only the diagnosis of BD. However, for human tissue donations, donors that had evolved into CRA are accepted. The number of corneal tissue donations has increased from donors who were given the diagnosis of BD and had evolved to CRA^[11,12]^. In the analysis of the profile of BVCH donors, it was observed that there is less family refusal in situations in which the potential donor was in CRA. It is assumed that most of the lay population believes that death occurs only with heartbeat cessation.

In a previous study conducted about survival^[4]^, between 1996 and 2004, BVCH received 1,041 hearts, with an average of 115 hearts/year. The progressive growth in the number of donations occurred because of the establishment of the National Transplant System in 1997. In the period of this study (2004 to 2014), BVCH received 2,149 hearts, with an average of 195 hearts/year. In the past seven years, Brazil presented a gradual and sustained growth in the number of organ and tissue donations, though below target^[13]^. Considering the number of organ retrievals by State, a disparity among them was noticed, which suggests a reflection not only of the teams' willingness and commitment, but also the activities of the States Transplant Centers.

It is essential that there be an interaction between the Organ and Tissue Banks and the State Centers of Organ Donation, promoting greater organ and tissue donation^[14]^. It is important that these Centers collaborate more effectively with the organ retrieval centers and health authorities, training the professionals involved in the process of screening and selecting donors and retrieving tissue. Furthermore, it is necessary to support studies in the development and implementation of programmes to raise awareness of the population about organ and tissue donation.

The results come closer to national data concerning the profile of organ and tissue donors, since each Tissue Bank has technical and legal standards to follow for selection and collection. There are also minimum and maximum ages of donors for each type of tissue; for example, the age of a heart donor for heart valves is similar to those presented, which points to a higher prevalence of 18-64 year olds^[15]^.

Concerning gender, there was a predominance of males, representing 65.7% of donors (n=1,411), and 60% of the effective organ and tissue donors in Brazil are male. The same pattern is found in Spain, world reference in organ and tissue retrieval, where 60.7% of donors are male^[16]^.

Trauma contributed highly to heart donation for heart valves. The results were similar to the literature, with a great prevalence of traumatic causes, reflecting the violence in today's society, and CVA as main cause of death^[17,18]^. Comparing the causes of death with gender and age of the donors, it was noted that trauma was the major cause of death of men aged between 16 and 35 years old, while CVA prevailed among women in the age group of 15 to 36 years old^[19,20]^.

## CONCLUSION

This study is presented as a pioneer for the country's transplant scenario, describing the profile of heart valve donors. The information offered can contribute to the evaluation and monitoring of the operation as well as the implementation and efficiency of the actions proposed by the transplant policy in national, state and local levels.

The lack of training of health professionals and of population knowledge about the organ donation process directly impact the number of donors and, consequently, the number of transplants. Thus, it is essential that the Tissue Banks work together with the CNCDs in developing professional training and permanent educational programs for awareness, education and encouragement of the population since the family's refusal is one of the main obstacles for effective organ and tissue donation.

As future work, it is recommended studies that approach issues such as tissue disposal in the pre and post-processing stages, qualified and released tissue for transplant, and tissue recipients'profile.

**Table:** 

Authors' roles & responsibilities	
RMF	Conception and study design; execution of operations and/or trials; analysis and/or data interpretation; final manuscript approval
MTBAC	Manuscript writing or critical review of its content; final manuscript approval
OCJ	Final manuscript approval
AMOSA	Analysis and/or data interpretation; statistical analysis; manuscript writing or critical review of its content; final manuscript approval

## References

[1] Brasil, Ministério da Saúde (2009). Portaria n.º 2.600/GM, de 21 de outubro de 2009. Aprova o
Regulamento Técnico do Sistema Nacional de Transplantes.

[2] Schiozer W (2012). Banco de pele no Brasil. Rev Bras Queimaduras.

[3] Costa F, Dohmen P, Vieira E, Lopes SV, Colatusso C, Pereira EWL (2007). Operação de Ross com homoenxertos valvares
decelularizados: resultados de médio prazo. Rev Bras Cir Cardiovasc.

[4] Costa MTBA, Costa FDA, Nazareno LCF, Domchoski J, Peruzzo AM, Colatusso C (2005). Análise das atividades dos oito anos iniciais do Banco de
Valvas Cardíacas Humanas do Hospital de Caridade da Irmandade da
Santa Casa de Misericórdia de Curitiba. Rev Bras Cir Cardiovasc.

[5] Brasil, Presidência da República (1997). Lei n.º 9.434, de 4 de fevereiro de 1997. Brasília.
Dispõe sobre a remoção de órgão, tecidos
e partes do corpo humano para fins de transplante.

[6] First MR (1992). Transplantation in the nineties. Transplantation.

[7] Heng WL, Seck T, Tay CP, Chua A, Song C, Lim CH (2013). Homograft banking in Singapore: two years of cardiovascular
tissue banking in Southeast Asia. Cell Tissue Bank.

[8] Jashari R, Van Hoeck B, Tabaku M, Vanderkelen A (2004). Banking of the human heart valves and the arteries at the
European homograft bank (EHB): overview of a 14-year activity in this
International Association in Brussels. Cell Tissue Bank.

[9] Shiratori CN, Hirai FE, Sato EH (2011). Características dos doadores de córneas do Banco de
Olhos de Cascavel: impacto do exame anti-HBc para hepatite B. Arq Bras Oftalmol.

[10] Rodrigues TB, Chagas MIO, Brito MCC, Sales DS, Silva RCC, Souza AMA (2013). Perfil de potenciais doadores de órgãos em hospital
de referência. Rev Rene.

[11] Paz ACAC (2001). Caracterização dos doadores de órgãos
e tecidos para transplante do Estado do Piauí, de 2000 a
2009. Enfermagem em Foco.

[12] Lavagnoli CF, Severino ES, Vilarinho KA, Silveira Filho LM, Oliveira PP, Petrucci O (2012). Associated factors with survivals in patients undergoing
orthotopic heart transplant using retrograde blood
microcardioplegia. Rev Bras Cir Cardiovasc.

[13] Associação Brasileira de Transplante de
Órgãos Entendendo a doação de órgãos, 2008.

[14] Morales Pedraza J, Lobo Gajiwala A, Martinez Pardo ME (2012). A review of the International Atomic Energy Agency (IAEA)
international standards for tissue banks. Cell Tissue Bank.

[15] Associação Brasileira de Transplante de
Órgãos (2009). Dimensionamento dos Transplantes no Brasil e em Cada Estado:
2007-2014. RBT.

[16] Organizacion Nacional de Trasplates (2009). Memoria de Actividad.

[17] Aguiar MIF, Araújo TOM, Cavalcante MMS, Chaves ES, Rolim ILTP (2010). Perfil de doadores efetivos de órgãos e tecidos no
Estado do Ceará. Rev Min Enferm.

[18] Mulinari LA, Navarro FB, Pimentel GK, Miyazaki SM, Binotto CN, Pelissari EC (2008). The use and midium-term evaluation of decellularized allograft
cusp in the surgical treatment of the tetralogy of Fallot. Rev Bras Cir Cardiovasc.

[19] Moraes EL, Silva LBB, Moraes TC, Paixão NCS, Izumi NMS, Guarino AJ (2009). O perfil de potenciais doadores de órgãos e
tecidos. Rev Latino-Am Enfermagem.

[20] Costa FDA, Fornazari DF, Matsuda CN, Torres RA, Sardetto E, Ferreira ADA (2006). Ten years experience of aortic valve replacement with aortic
homograft root replacement. Braz J Cardiovasc Surg.

